# Electron transport in a GaPSb film

**DOI:** 10.1186/1556-276X-7-640

**Published:** 2012-11-23

**Authors:** Shun-Tsung Lo, Hung En Lin, Shu-Wei Wang, Huang-De Lin, Yu-Chung Chin, Hao-Hsiung Lin, Jheng-Cyuan Lin, Chi-Te Liang

**Affiliations:** 1 Graduate Institute of Applied Physics, National Taiwan University, Taipei, 106, Taiwan; 2 Department of Physics, National Taiwan University, Taipei, 106, Taiwan; 3 Graduate Institute of Electronics Engineering, National Taiwan University, Taipei , 106, Taiwan; 4 Department of Electrical Engineering, National Taiwan University, Taipei, 106, Taiwan; 5 Electronics Testing Center, No. 8, Lane 29, Guishan Shiang, Taoyuan County, Taiwan, 333

**Keywords:** Mott variable range hopping, GaPSb, GaAs

## Abstract

We have performed transport measurements on a gallium phosphide antimonide (GaPSb) film grown on GaAs. At low temperatures (*T*), transport is governed by three-dimensional Mott variable range hopping (VRH) due to strong localization. Therefore, electron–electron interactions are not significant in GaPSb. With increasing *T*, the coexistence of VRH conduction and the activated behavior with a gap of 20 meV is found. The fact that the measured gap is comparable to the thermal broadening at room temperature (approximately 25 meV) demonstrates that electrons can be thermally activated in an intrinsic GaPSb film. Moreover, the observed carrier density dependence on temperature also supports the coexistence of VRH and the activated behavior. It is shown that the carriers are delocalized either with increasing temperature or magnetic field in GaPSb. Our new experimental results provide important information regarding GaPSb which may well lay the foundation for possible GaPSb-based device applications such as in high-electron-mobility transistor and heterojunction bipolar transistors.

## Background

III-V-based alloys and heterostructures have been attracting much interest because of their great device applications as well as their fundamental importance. A major issue of composing III-V-based systems is the miscibility gap in solids [1]. It is known that the mixing enthalpy of the systems, such as GaAs-GaSb and GaP-GaSb, is proportional to the square of the difference in lattice constant of the two end binary components of the system [2]. This reason prohibits the epitaxial growth of most alloys at ordinary growth temperatures [3]. Therefore, the epitaxial growth of these systems was not achieved until the first growth of GaAs-GaSb was done in 1979 by carrying out the growth under a high-supersaturation condition, such as molecular beam epitaxy (MBE) [4]. The ternary alloy gallium phosphide antimonide (GaP_1−*x*_Sb_*x*_) was grown on GaAs for the first time using the organometallic vapor-phase epitaxy method in 1988 [5]. Since then, there has been a lack of work on gallium phosphide antimonide as well as on the transport behavior in such material.

For an intrinsic semiconductor, it usually shows insulating behavior in the sense that the resistance decreases with increasing temperature. At low temperatures (*T*), when the thermal energy is not high enough to excite carriers to the conduction band, transport is mostly dominated by variable range hopping (VRH) [6,7], indicating the strong localization of carriers. In the Mott VRH regime, the temperature dependence of the longitudinal resistance *R*_xx_ is of the form

(1) $$R_{\text{xx}}=R_{0}\text{exp}\left[{\left(T_{0}/T\right)}^{p}\right]\text{,}$$

with the exponent *p* = 1/3 for two-dimensional (2D) systems or *p* = 1/4 for three-dimensional (3D) ones. Here, *R*_0_ is a prefactor and *T*_0_ is the characteristic temperature related to the localization length. When considering interactions, the suppression of density of states near the Fermi energy would lead to *p* = 1/2 for both 2D and 3D systems in Equation 1, known as Efros-Shklovskii VRH. Another important phenomenon in the strong localization regime is referred to as negative magnetoresistance (NMR) which results from the suppression of quantum interference between forward scattering hopping paths as the magnetic field (*B*) is applied [8-11]. On the other hand, at high *B* where the hopping probability between different sites is significantly reduced due to the shrinkage of wave function, positive magnetoresistance (PMR) occurs [12]. NMR and PMR refer to the case where the resistance decreases and increases, respectively, with increasing *B*.

At elevated *T*, activation conduction can contribute to the transport as well, which is usually observed in a doped semiconductor like a Si delta-doped GaAs. In an n-type semiconductor, the Fermi energy is shifted towards the conduction band edge from the mid-gap position. In this activation regime, the resistance as a function of *T* can be described by

(2) $$R_{\text{xx}}=R_{a}\text{exp}\left(E_{\text{a}}/k_{\text{B}}T\right)$$

with Boltzmann constant (*k*_B_), activation energy (*E*_a_), and a prefactor (*R*_a_). Such a crossover from VRH conduction to an activation one with increasing *T* has already been observed in Si delta-doped GaAs grown by MBE [13].

Since there is a dearth of work on the transport properties of GaPSb, important physical phenomena such as the type of carriers, the strength of carrier-carrier interactions, transport behavior, and so on require further investigations. In this work, we report extensive transport studies of a GaPSb film grown on a GaAs substrate. Such a device is fully compatible with the existing GaAs-based high-electron-mobility transistor (HEMT) technology. Moreover, the GaPSb-based material system may well be of great device applications in heterojunction bipolar transistor (HBT), high-power devices, and nanoelectronics [14-16]. We shall show that the carriers in GaPSb are electrons. Moreover, at low temperatures, electrons in GaPSb are strongly localized and can be described by 3D Mott VRH. Therefore, electron–electron interactions are negligible in GaPSb. Furthermore, we show that VRH and activation conduction can coexist, which is consistent with the observed peculiar *T* dependence of *n*. The measured gap from the observed activated behavior (approximately 20 meV) is comparable to thermal broadening at room temperature (approximately 25 meV). Such a result suggests that electrons are delocalized in nominally undoped GaPSb at room temperature. Our new experimental results provide important information for possible device applications as well as modeling using the GaPSb-based materials.

## Methods

The undoped 720-nm-thick GaP_0.71_Sb_0.29_ was grown on a 4-in. (100) 2° off-axis toward (110) GaAs substrate by an Aixtron 2600G3 (Aixtron SE, Aachen, Germany) metal organic chemical vapor deposition, using trimethygallium and trimethyantimony as metal organic sources and phosphine (PH_3_) and arsine (AsH_3_) as hydride sources. The growth conditions for the GaP_0.71_Sb_0.29_ are similar to those published in [17]. We used Vegard's law to determine the averaged Sb composition in this GaP_0.71_Sb_0.29_ bulk sample by X-ray diffraction. The reactor was heated up to 700°C to clean the substrate surface with AsH_3_ before the epitaxial growth and then cooled down to 530°C for the GaPSb growth. The V/III ratio and growth rate were approximately 1 and 1 μm/h, respectively. On top of the GaP_0.71_Sb_0.29_ film, a 20-nm GaAs cap was deposited. The Hall bar device was fabricated by standard photolithography and etched by a top-down process with a solution at the mixture ratio of H_3_PO_4_/H_2_O_2_/H_2_O = 1:1:10. Four-terminal measurements were performed by standard dc techniques in a top-loading He^3^ cryostat.

## Results and discussion

**Figure 1:**
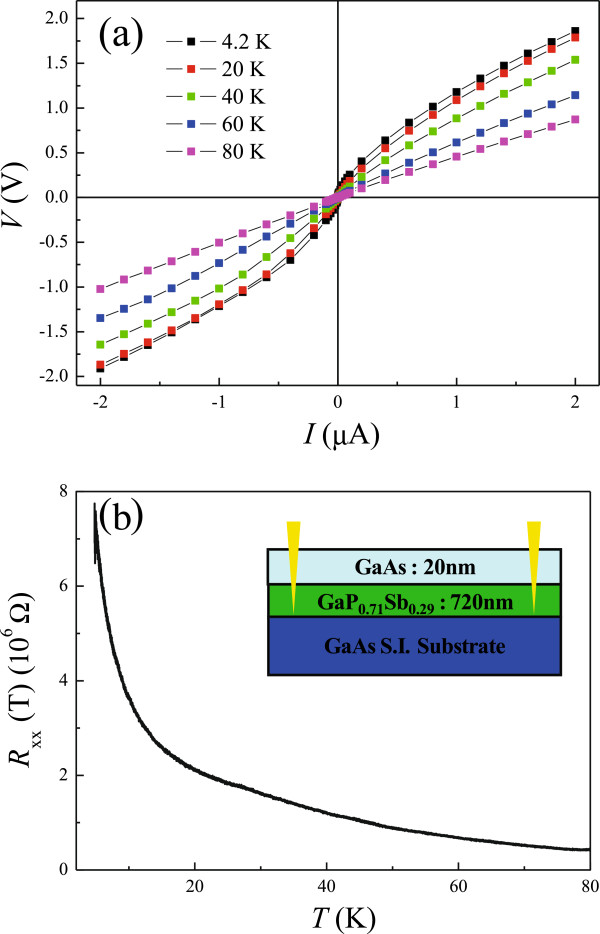
**The current–voltage measurements at various temperatures and the longitudinal resistance. (a)** Four-terminal current–voltage measurements *V*(*I*) at various temperatures *T*. **(b)** Longitudinal resistance *R*_xx_ as a function of *T*. The inset shows the sample structure.

Figure 1a shows the current–voltage measurements *V*(*I*) at various temperatures. For *T* ≦ 20 K, *V*(*I*) is highly nonlinear, suggesting that the carriers are strongly localized at low temperatures. With increasing *T* above 60 K, it shows ohmic behavior over the whole applied current range −2 μA ≦ *I* ≦ 2 μA. The longitudinal resistance *R*_xx_ as a function of *T* is measured in the ohmic regime with a current *I* = 1 nA, and the result is shown in Figure 1b. The inset in Figure 1b illustrates the structure of the device.

**Figure 2:**
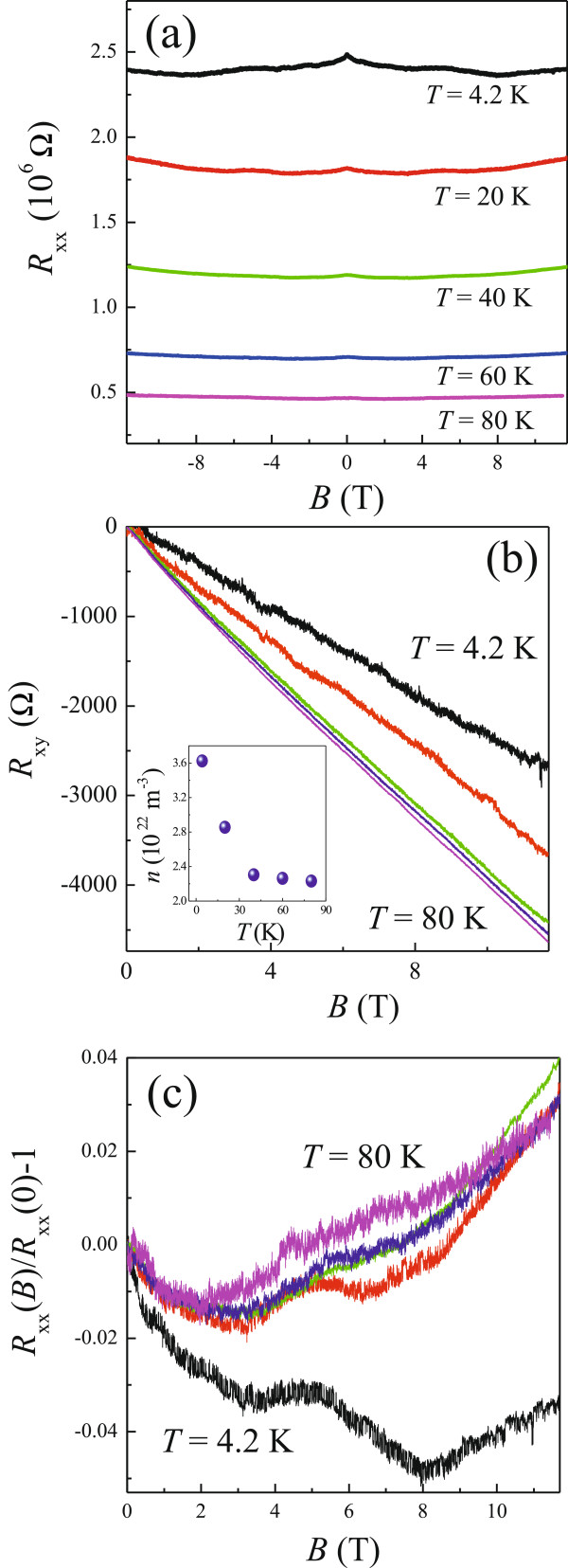
**The longitudinal and Hall resistances as a function of *B *at various *T. *(a)** The longitudinal (*R*_xx_) and **(b)** Hall resistances (*R*_xy_) as a function of *B* at various *T*. From top to bottom: *T* = 4.2, 20, 40, 60, and 80 K, respectively. The inset to (b) shows the carrier concentration *n* as a function of *T* determined by the Hall slope. **(c)***R*_xx_(*B*)/*R*_xx_(0)−1 against *B*.

In order to further study the transport mechanism in such a highly disordered system, the longitudinal and Hall resistances (*R*_xx_ and *R*_xy_) as a function of *B* at various *T* were measured, and the results are presented in Figure 2a,b, respectively. A current of 100 nA was applied in magnetotransport measurements so as to improve signal-to-noise ratio. As shown in Figure 2a, *R*_xx_(*B*) behaves as an insulator over the whole measurement range. For clarity, we plot *R*_xx_(*B*)/*R*_xx_(0)−1 against *B* in Figure 2c. With increasing *B*, a slight decrease of resistance, characteristic of NMR, and subsequent increase of it, characteristic of PMR, can be observed at *T* = 4.2 K.

**Figure 3:**
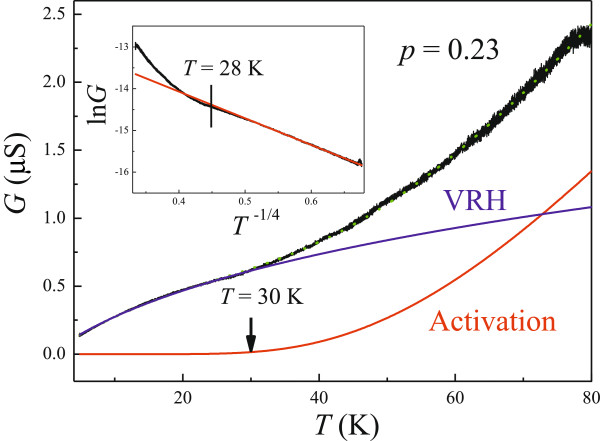
***V*(*I*) measurements at various *B *ranging from *B *= 0 to *B *= 4 T in steps of 0.4 at *T *= 4.2 K.**

Figure 2b shows the Hall resistance which is always negative, indicating that electrons are the major carriers in GaPSb. The Hall slope *R*_H_ = −*R*_xy_/*B* = 1/(*ned*) increases with increasing *T*, and therefore, the estimated carrier concentration *n* decreases with increasing *T*, as shown in the inset of Figure 2b, where *d* is the thickness of the measured GaPSb film. It can be observed that *n* changes rapidly for *T* < 40 K, while only a slight decrease of *n* can be seen at higher *T*. Neither activation of carriers to the conduction band nor electron–electron interaction effects can explain such results as both would tend to decrease the Hall slope with increasing *T*[18]. As suggested by our previous study on a Si delta-doped GaAs single quantum well [19] and the work performed by Yildiz et al. on Si delta-doped GaAs [13], the observation of non-monotonic *T* dependence of *n*, that is, it first decreases and then increases with increasing *T*, can be regarded as a piece of evidence that there is a crossover from VRH conduction to the activation one with increasing *T* since the VRH conduction would affect the estimation of Hall slope at low *T* and results in a reduction of *n*. According to our results, the decrease of *n* with increasing *T* as shown in the inset of Figure 2b, we are able to deduce that the VRH conduction coexists with the activation one over the whole measurement range. To further support this argument, the two-band conduction model [20-22] is used to analyze the *R*(*T*) results shown in Figure 1a. In this model, one can expect that the longitudinal conductance *G*_xx_ = 1/*R*_xx_ is determined as the combination of VRH and activation conduction and is described by

(3) $$G_{\text{xx}}\left(T\right)=G_{\text{c}}\text{exp}\left(-E_{\text{a}}/kT\right)+G_{0}\text{exp}\left(-{\left(T_{0}/T\right)}^{p}\right)$$

with prefactors *G*_c_ and *G*_0_[23]. The other parameters are the same as those used in Equations 1 and 2. As shown by the dotted line in Figure 3, there is a good fit to Equation 3, and the parameters are obtained as *G*_c_ = 20 μS, *E*_a_ = 20 meV, *G*_0_ = 10 μS, *T*_0_ = 1,409 K, and *p* = 0.23. The determined exponent *p* = 0.23 close to the expected value of 0.25 for 3D Mott VRH demonstrates that our GaPSb film is intrinsically three-dimensional and the interactions between electrons is insignificant. Moreover, a transport gap of *E*_a_ = 20 meV is found, which is very useful in the fabrication of heterojunction devices since the thermal energy is enough to activate electrons in GaPSb at room temperature, an attractive property for possible HEMT and HBT applications. In the 3D Mott VRH model, *k*_B_*T*_0_ = 18/(*N*(*E*_F_)*ξ*^3^ such that *ξ* = (18/*k*_*B*_*T*_0_/*N*(*E*_*F*_))^1/3^. With the obtained *T*_0_ ~ 1,409 K and $N_{3D}\left(E_{F}\right)=\frac{m^{*}}{\pi \hbar^{2}d}=\frac{9.11\cdot 10^{-31}}{\pi \hbar^{2}720\cdot 10^{-9}}=3.62\cdot 10^{43}m^{-3}$ under the assumption that $N_{3D}\left(E_{F}\right)=\frac{N_{2D}\left(E_{F}\right)}{d}$ and m^*^ = *m*_e_, *ξ* = 29.5 nm, *R*_hop_ ~ 29.5(1,409/*T*)^1/4^ nm and the hoping energy *w*_hop_ ~ *k*_B_*T*(1,409/*T*)^1/4^ are obtained. Hence, for 4.2 K ≤ *T* ≤ 80 K, 60 nm ≤ *R*_hop_ ≤ 126 nm, and 1.55 meV <*w*_hop_ < 14 meV. From these estimations showing *R*_hop_ << sample size and *w*_hop_<*E*_a_ ~ 20 meV over the temperature range of interest, we can learn that VRH transport indeed plays an important role in our system. If the nearest-neighbor-hopping (NNH) conduction plays a role, *p* ~ 1 should be given. However, we show that *p* ~ 0.23. Moreover, from the observed unusual temperature dependence of the carrier density, it is expected that the activation conduction would contribute to the conduction at high-enough temperatures. Therefore, we believe that the high-*T*-activated behavior is predominantly caused by a thermal activation conduction process rather than NNH.

**Figure 4:**
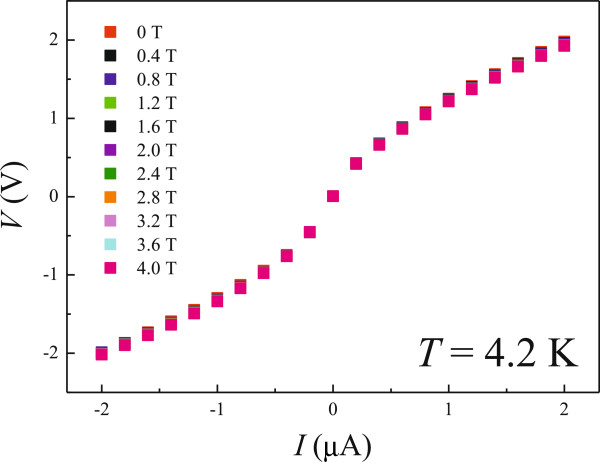
***R***_**xx**_**(*B *= 0) as a function of *T *ranging from 4.2 to 80 K (black curve).** The blue solid line denotes the theoretical VRH contribution described by Equation 3 and the red one corresponds to the activation part. Their sum is presented by the green dotted line. The inset shows ln*G* as a function of *T*^−1/4^.

After substituting the obtained parameters into Equation 3, the contributions of VRH and activation conduction are shown separately in Figure 4. The blue line denotes the VRH part, and the red one is for the activation conduction in Equation 3. It can be seen clearly that VRH dominate the transport for *T* < 30 K, consistent with the observations of nonlinear *V*(*I*) and the rapid decrease of *n* at low temperatures, which suggests that the carriers are indeed strongly localized at low *T*. The deviation from the 3D Mott VRH at high *T* can be observed clearly by plotting ln*G* as a function of *T*^−1/4^, which can be seen in the inset of Figure 4. With increasing *T*, the activation contribution can compete with the VRH one, and therefore the ohmic behavior can be restored at high *T*, which can be seen in Figure 1a. Such results demonstrate that the carriers are delocalized with increasing *T*.

For carriers in the conduction band, diffusive motion can dominate the transport, in which nonlinear *V*(*I*) does not occur. As inferred from the NMR shown in Figure 2a, delocalization of carriers occurs with increasing *B* as well. However, as known from the nonlinear *V*(*I*) at various *B* for *T* = 4.2 K shown in Figure 3, the carriers are still strongly localized. Therefore, the observed NMR should result from the quantum interference feature in the strong localization regime, which is different from the *T*-induced delocalization process.

## Conclusion

We have performed extensive transport measurements on a GaPSb film grown by MOCVD. At a low *T*, variable range hopping dominates the transport, and then, a crossover from VRH to activation conduction occurs with increasing *T*. In the intermediate temperature range, coexistence of the VRH and activation conduction can be found, consistent with the observation of peculiar *T* dependence of carrier concentration. The observed nonlinear current–voltage relations further support that the carriers are strongly localized at low temperatures. The carriers can be delocalized either with increasing *T* or increasing *B*. The measured transport gap of approximately 20 meV is comparable to thermal broadening at room temperature (approximately 25 meV). Therefore, our results show that electrons can be thermally activated even in an intrinsic GaSbP film at room temperature. This interesting result may find applications in designing a transistor with a GaPSb base. Moreover, since our GaPSb film is grown on GaAs, such a device is fully compatible with existing GaAs-based HEMT technology. Our work may be useful for possible applications such as HEMT and HBT devices based on the GaPSb material system.

## Competing interests

The authors declare that they have no competing interests.

## Authors’ contributions

STL, HEL, SWW, and JCL performed the measurements. STL and CTL drafted the paper. HDL proposed the theoretical models. HHL coordinated the project. YCC prepared the sample. All authors read and approved the final version of the manuscript.

## Authors’ information

STL obtained his B.Sc. degree at National Taiwan University (NTU) in 2010 and is pursuing his Ph.D. degree at the Graduate Institute of Applied Physics, NTU. He won the Dr. An-Tai Chen Scholarship, Mr. Ming Kao Scholarship, and Creative Award for college students participating in special research project provided by the NSC in 2009. HEL obtained his master's degree at NTU in 2011. SWW obtained his master's degree at NTU in 2011. He won the prestigious Lam Research Award in 2012. HDL obtained his B.S. degree at Chinese Culture University, Taiwan, and his Ph.D. degree at Mississippi State University, USA, and currently works as a project engineer at the Electronics Testing Center, Tao-Yuan, Taiwan (ROC). YCC received his B.S. degree in physics from NTU in 1996 and his MSEE degree from National Chiao-Tung University (NCTU) in 2000. He currently is a Ph.D. student studying at the Graduate Institute of Electronics Engineering, NTU. HHL obtained his BSEE, MSEE, and Ph.D. degree at NTU and is currently a professor of the Department of Electrical Engineering, NTU. JCL obtained his master's degree at NTU in 2012. CTL obtained his B.Sc. degree at NTU in 1990 and his Ph.D. degree in physics at Cambridge University, UK, in 1996 and is currently a professor of physics at NTU. He is also a topical editor for *Current Applied Physics* and an associate editor for the *Journal of Nanoscience Letters*.
